# The chromatin and single-cell transcriptional landscapes of CD4 T cells in inflammatory bowel disease link risk loci with a proinflammatory Th17 cell population

**DOI:** 10.3389/fimmu.2023.1161901

**Published:** 2023-08-03

**Authors:** Tiago S. Medina, Alex Murison, Michelle Smith, Gabriela S. Kinker, Ankur Chakravarthy, Glauco A. F. Vitiello, Williams Turpin, Shu Yi Shen, Helen L. Yau, Olga F. Sarmento, William Faubion, Mathieu Lupien, Mark S. Silverberg, Cheryl H. Arrowsmith, Daniel D. De Carvalho

**Affiliations:** ^1^ Princess Margaret Cancer Centre, University Health Network, Toronto, ON, Canada; ^2^ International Research Center, A.C. Camargo Cancer Center, São Paulo, Brazil; ^3^ Division of Gastroenterology, Mount Sinai Hospital, University of Toronto, Toronto, ON, Canada; ^4^ Department of Medical Biophysics, University of Toronto, Toronto, ON, Canada; ^5^ Division of Gastroenterology and Hepatology, Mayo Clinic, Rochester, MN, United States; ^6^ Structural Genomics Consortium, University of Toronto, Toronto, ON, Canada

**Keywords:** inflammatory bowel disease (IBD), CD4 T cells, genetic variants, pathogenic (p)Th17 cells, cytotoxicity, Crohn's disease, Ulcerative colitis

## Abstract

**Introduction:**

The imbalance between Th17 and regulatory T cells in inflammatory bowel diseases (IBD) promotes intestinal epithelial cell damage. In this scenario, T helper cell lineage commitment is accompanied by dynamic changes to the chromatin that facilitate or repress gene expression.

**Methods:**

Here, we characterized the chromatin landscape and heterogeneity of intestinal and peripheral CD4 T cellsfrom IBD patients using in house ATAC-Seq and single cell RNA-Seq libraries.

**Results:**

We show that chromatin accessibility profiles of CD4 T cells from inflamed intestinal biopsies relate to genes associated with a network of inflammatory processes. After integrating the chromatin profiles of tissue-derived CD4 T cells and in-vitro polarized CD4 T cell subpopulations, we found that the chromatin accessibility changes of CD4 T cells were associated with a higher predominance of pathogenic Th17 cells (pTh17 cells) in inflamed biopsies. In addition, IBD risk loci in CD4 T cells were colocalized with accessible chromatin changes near pTh17-related genes, as shown in intronic STAT3 and IL23R regions enriched in areas of active intestinal inflammation. Moreover, single cell RNA-Seq analysis revealed a population of pTh17 cells that co-expresses Th1 and cytotoxic transcriptional programs associated with IBD severity.

**Discussion:**

Altogether, we show that cytotoxic pTh17 cells were specifically associated with IBD genetic variants and linked to intestinal inflammation of IBD patients.

## Introduction

The inflammatory bowel diseases (IBD) are a group of chronic intestinal inflammatory disorders that include ulcerative colitis (UC) and Crohn’s disease (CD) (1). UC is a condition that primarily affects the colon with a continuous inflammatory process beginning in the rectum and extending proximally, whereas CD can affect the small or large intestine and the inflammatory pattern may be patchy with normal and affected areas immediately adjacent to one another. These diseases commonly result in ulceration of the intestine and clinically cause abdominal pain, diarrhea and intestinal bleeding (2). Through large genome-wide association studies, the genetic architecture of IBD has been well characterized with over 200 susceptibility loci identified (3–8). However, these disorders are rapidly increasing in incidence and prevalence, particularly in developing countries, suggesting a strong environmental contribution (9). Moreover, for most risk loci identified, there remains a gap in understanding the mechanism by which intestinal inflammation occurs in affected individuals (10, 11).

In IBD, dysregulated CD4 T cells are critical drivers of intestinal inflammation (12, 13). Due to the complexity of the CD4 T cell response, it has been challenging to determine what dysregulated T cell subpopulation is the major driver of intestinal inflammation in IBD (12–14). In addition, the inflammatory cascade driven by dysregulated intestinal CD4 T cells is variable and intrinsically dependent on host genetics and environmental triggers. Further, CD4 T cell fate is extensively shaped by factors that affect specific signaling pathways in the tissue microenvironment (15). For instance, upon TGF-β1 signaling pathway activation, effector CD4 T cells give rise to T regulatory cells (Tregs) (16). Inclusion of IL-6 in the Treg-specific conditions prevents Treg cell development and promotes the differentiation of Th17 cells (17, 18). However, evidence suggests that Th17 cells are further characterized by their inflammatory potential as either regulatory Th17 (rTh17) cells or pathogenic Th17 (pTh17) cells (19–21). TGF-β1 is a critical factor that contributes to Th17 cell fate, as its activity can regulate Th17 cell balance between pro- and anti-inflammatory subpopulations. Thus, TGF-β1 and IL-6 signaling pathways induce a regulatory network that polarizes Th17 cells towards a suppressive profile (rTh17 cells), whereas a TGF-β1-independent differentiation governed by IL-6, IL-1 and IL-23 induces a CD4 T cell pro-inflammatory profile (pTh17 cells) that aggravates immune-mediated diseases (19–22). This has established a framework for interpreting IBD risk variants in the context of CD4 T cell plasticity.

Since host genetic variants have a crucial impact on shaping the immune response that leads to IBD immunopathogenesis (8), genetic perturbations that alter the balance between Th17 effector cells and T regulatory cells and favor gut inflammation have been implicated in the pathogenesis of both UC and CD (23). IBD risk loci play a role in several inflammatory pathways (3, 23, 24), including regulation of the adaptive immune response (25). Indeed, several IBD risk loci can modulate T cell function, with at least 24 variants being implicated within the Th17 cell regulatory network, such as *STAT3*, *IL23R*, *CCR6*, *AHR* and *CCL2* (3, 24, 26). In addition, SNPs in genes associated with Treg cell differentiation (i.e, *SMAD3*, *SMAD7*, *TNFRSF18*, *IL2RA*, *IL10*) have also been linked to IBD (10, 25). Despite this progress, the precise mechanisms for how IBD risk loci influence the CD4 T cell response is largely unknown. Since the differentiation of naïve CD4 T cells into one of several effector lineages is a process that is fundamentally epigenetic in nature and involves remodeling of the transcriptional program through lineage-dependent transcription factors (TF) (27), we hypothesized that genetic and epigenetic factors contribute to the establishment of intestinal inflammation in IBD patients. As such, the assessment of epigenetic factors and genetic risk variants as inductors of CD4 T cell heterogeneity and complexity is critical to understanding how T cell subsets contribute to the onset of intestinal inflammation and to identify strategies that target such inflammatory subpopulations. Here, we undertook a characterization of the chromatin accessibility and gene expression profiles of CD4 T cells to assess their contribution to inflammation of the gut and showed that IBD risk loci are linked to Th17 cells and that a pTh17 cell subpopulation co-expressing Th1 and cytotoxic gene programs is associated with intestinal inflammation in IBD patients.

## Materials and methods

### Patient recruitment and sample collection

IBD patients were recruited during regularly scheduled endoscopic follow-up, at Mount Sinai Hospital (MSH) in Toronto, Canada, in accordance with approval granted by the hospital’s Research Ethics Board (University Health Network REB# 15-9499.2). Written informed consents were received from all patients and healthy volunteers prior to the enrollment in this study.

Following patient recruitment, demographic and clinical data were obtained from all IBD patients. Blood was obtained from consented subjects. Biopsies were taken using standard forceps, from the terminal ileum or sigmoid colon. During the endoscopy, physicians documented the appearance of the biopsy sites using standardized endoscopic scoring indices for that segment. For a subset of individuals, matched samples were taken from a location based on identifying a region where inflamed and non-inflamed tissue were found in the same segment or in close proximity. Non-inflamed biopsy samples were taken from a region with a Mayo Score of 0 for UC or a Simple Endoscopic Score for Crohn’s Disease (SES-CD) of 0 for CD. Inflamed biopsy samples were taken from regions with a Mayo score of 2 or greater or a SES-CD score of 3 or greater for UC and CD, respectively. Blood samples were also obtained for preparation of ATAC libraries from consented healthy subjects. Clinical characteristics of patients and healthy donors are provided in **Tables S1–S5**.

### Reagents and antibodies

For *in vitro* polarization assays, we used recombinant human IL-12 [20ng/mL], IL-4 [20ng/mL], IL-23 [10ng/mL], IL-6 [20ng/mL], TGF-β1 [10ng/mL] and IL-1β [10ng/mL] cytokines (all purchased from R&D Systems) and neutralizing human IFN-γ [10g/mL], IL-4 [10g/mL], IL-23p19 [10g/mL], IL-6 [10g/mL], TGF-β [10g/mL], and IL-1 [10g/mL] antibodies (all purchased from R&D Systems). For sorting of live gut-resident CD4 T cells used in ATAC-Seq experiments, we stained cells with Sytox-Blue Dead Cell Stain (Invitrogen – Cat. Number S34857), APC anti-human CD3 (BD Biosciences – Cat. Number 555335), FITC anti-human CD4 (Biolegend – Clone RPA-T4) and PE anti-human CD8 (BD Biosciences – Cat. Number 555635) antibodies. For single cell RNA-Seq experiments, we used the same antibodies described for ATAC along with APC/Cy7 anti-human CD161 (Biolegend – Clone HP-3G10) and PerCP/Cy5.5 anti-human TCR V24-J18 (Biolegend – Clone 6B11) antibodies.

### *In vitro* CD4 T cell polarization assays

For CD4 T cell polarization, naive CD4 T cells were first isolated using Naive CD4+ T cell Isolation Kit (Miltenyi) and immediately activated with dynabeads human T-activator anti-CD3/CD28 (Thermo Fisher Scientific) under Th1, Th2, pTh17, rTh17, and Treg conditions. For Th1 polarization, we cultured human CD4 T cells in the presence of recombinant human IL-12 [20ng/mL] and neutralizing human IL-4 [10μg/mL], IL-23p19 [10μg/mL], IL-6 [10μg/mL], TGF-β [10μg/mL], and IL-1β [10μg/mL] antibodies added at day 0. For Th2 polarization, we cultured CD4 T cells in the presence of recombinant human IL-4 [20ng/mL] and neutralizing human IFN-γ [10μg/mL], IL-23p19 [10μg/mL], IL-6 [10μg/mL], TGF-β [10μg/mL], and IL-1β [10μg/mL] antibodies added at day 0. For Treg-like polarization, we cultured CD4 T cells in the presence of recombinant human TGF-β1 [10ng/mL] and neutralizing human IFN-γ [10μg/mL], IL-23p19 [10μg/mL], IL-6 [10μg/mL], IL-4 [10μg/mL], and IL-1β [10μg/mL] antibodies added at day 0. For rTh17 polarization, we cultured CD4 T cells in the presence of the recombinant human cytokines TGF-β1 [10ng/mL] and IL-6 [20ng/mL] plus neutralizing human IFN-γ [10μg/mL] and IL-4 [10μg/mL] antibodies added at day 0. For pTh17 polarization, we cultured CD4 T cells in the presence of the recombinant human cytokines IL-6 [20ng/mL], IL-1β [10ng/mL] and IL-23 [10ng/mL] plus neutralizing human IFN-γ [10μg/mL], IL-4 [10μg/mL] and TGF-β [10μg/mL] antibodies added at day 0. At day 4, forty thousand cells were used to perform ATAC-Seq on CD4 T cell subtypes. At day 5, the remaining cells were used to confirm by flow cytometry that the cells were indeed polarized to their corresponding subpopulations, by staining intracellularly the key cytokine(s) of each CD4 T cell subtype.

### ATAC-Seq of biopsies and blood samples

To determine the chromatin accessibility of CD4 T cells from intestinal biopsies and blood samples (**Tables S1, S2**), Assay for Transposase-Accessible Chromatin with high-throughput sequencing (ATAC-Seq) was performed. Eleven inflamed and eighteen non-inflamed biopsies from UC or CD patients were collected for ATAC-Seq. For 6 patients, we had matched inflamed and non-inflamed biopsies from the same individual. Two thousand live CD4 T cells were used to determine the chromatin accessibility of the cells. ATAC-Seq was also performed on blood samples of 9 age- and gender-matched healthy subjects (University Health Network REB# 11-0343) and 25 IBD patients.

Briefly, biopsies were collected fresh in the endoscopy unit in RPMI 1640 supplemented with 10% fetal bovine serum (FBS) and 1% penicillin/streptomycin and were immediately washed and digested for 45 minutes at 37°C with collagenase from *Clostridium histolyticum* (Sigma). Fc receptors were blocked using Human BD Fc Block (BD Biosciences – Cat number 564220) for 15 minutes, and live CD8^-^CD4^+^CD3^+^ cells were stained for 30 minutes and sorted. For blood samples, live CD4 T cells were isolated using a magnetic cell isolation kit (Myltenyi Biotec).

We also performed ATAC-Seq on peripheral CD4 T cells of healthy subjects (**Table S3**) to generate ATAC signatures of CD4 T cell subpopulations. Briefly, blood samples were taken from 4 age-matched healthy volunteers (two males and two females), and naïve CD4 T cells were isolated using the naïve CD4^+^ T Cell Isolation Kit II, human (Miltenyi Biotec). Two hundred thousand cells were plated in a 96-well plate, activated with Dynabeads Human T-activator CD3/CD28 (bead-to-cell ratio of 1:1, as recommended by the manufacturer) (ThermoFisher Scientific) and polarized towards CD4 T cell subtypes with specific cocktails of recombinant cytokines and neutralizing antibodies for each subset for 5 days (as described in the previous section).

CD4 T cells were washed with PBS and lysed for 5 minutes on ice using a lysis buffer containing 10mM Tris-HCl, pH7.4, 10mM NaCl, 3mM MgCl_2_, 0.1% IGEPAL CA-630. After isolating crude nuclei, cells were treated for 30 minutes at 37°C with Tagment DNA buffer and Tagment DNA Enzyme (Nextera DNA Library Prep Kit, Illumina), which binds to the open areas of the chromatin, and then the DNA was purified by MinElute PCR Purification Kit (Qiagen). Transposed DNA fragments were amplified using specific adapters followed by purification with MinElute PCR Purification Kit (Qiagen). Fragments from 240-360pb were selected in the PippinHT system (Sage Science). The quality of the library and its DNA concentration were assessed by Bioanalyzer instruments (Agilent Technologies) and ultimately submitted for sequencing using Illumina HiSeq 2500 sequencer, V4 chemistry. Sequencing was done at Princess Margaret Genomics Centre (PMGC).

### Analysis of ATAC-Seq data

ATAC-Seq reads were trimmed and filtered for quality and then aligned to hg38 using Bowtie. Peaks were identified using MACS. EdgeR (28) was used to normalize the counts matrix and perform differential accessibility analysis between all relevant comparisons. Gene-to-peaks associations were defined when a peak mapped to the promoter (2.5 kb upstream to 0.5kb downstream of RefSeq TSS) or an exon of a given gene. For distal peaks, we applied the method used by Thurman et al. (29) (**Tables S6–S8**). Tracks were visualized using Integrative Genomics Viewer (Broad Institute). Motif-enrichment analysis was performed using HOMER with default settings (**Table S9**).

### Single cell RNA-Seq of biopsy samples

We additionally recruited 3 subjects with CD (**Tables S4, 5**) to assess the heterogeneity of CD4 T cells from inflamed and non-inflamed intestinal biopsies using single-cell RNA-Sequencing (scRNA-Seq). We collected matched non-inflamed and inflamed biopsy samples from the patients with CD. The *a priori* criteria to select the subjects for the scRNA-Seq experiments was for patients with established CD, female, Caucasian, between 20-40 years of age, and with no current medication or prior surgery. We also selected patients with colonic CD with inflamed and non-inflamed areas within the same colonic segment. These criteria were established in advance to reduce population and disease-related heterogeneity in the scRNA-Seq studies.

CD161^-^Vα24Jα18^-^CD8^-^CD4^+^ T cells were isolated from 3 inflamed and 3 non-inflamed biopsies of CD patients (**Figure S1**). The surface markers CD161 and Vα24Jα18 were used to exclude possible contamination with NK and NKT cells. Approximately 1,000 cells of each biopsy were captured and encapsulated before cDNA amplification using the 10X Genomics Chromium Platform. Samples were prepared as outlined by 10X Genomics Single Cell 3’ Reagent Kits v2 user guide. Briefly, samples were washed in PBS/0.04% BSA and re-suspended in 34μl of PBS + 0.04% BSA. The cell suspension was loaded onto the 10X single cell A chip. After droplet generation, samples were transferred onto a pre-chilled 96-well plate, heat sealed and incubated overnight in a Veriti 96-well thermal cycler (Thermo Fisher) for the reverse transcriptase (RT) reaction. Following the RT reaction, cDNA was recovered using the Recovery Agent provided by 10X Genomics and subsequently cleaned up using a Silane DynaBead (Thermo Fisher) mix as outlined by the user guide. Purified cDNA was amplified for 14 cycles before being cleaned up using SPRIselect beads (Beckman). Samples were diluted 4:1 (elution buffer (Qiagen):cDNA) and run on a Bioanalyzer (Agilent Technologies) to determine cDNA concentration. cDNA libraries were prepared as outlined by the Single Cell 3’ Reagent Kits v2 user guide with modifications to the PCR cycles based on the calculated cDNA concentration. Samples were sequenced on a HiSeq 2500 with the following run parameters: Read 1 – 26 cycles, read 2 – 98 cycles, index 1 – 8 cycles.

### Analysis of scRNA-Seq data

Raw sequencing data were pre-processed using the CellRanger pipeline (10X Genomics) to yield filtered count matrices for cells from the three patients profiled. Data were scaled with *computeSumFactors* from Scran, log(scaled + 1) transformed, and batch corrected with Scanorama using a set of consensus genes with highly variable expression in all samples. We excluded low quality cells with less than 200 genes detected. Clustering analysis was performed using *RunPCA, FindNeighbors, FindClusters* from Seurat (30). Cell clusters were visualized using the uniform manifold approximation and projection (UMAP) dimensional reduction. We identified differentially expressed genes (log2(fold change) > 0.5 or < -0.5, adjusted p < 0.05) by cells in each cluster, compared to all other cells, using the two-sided t test implemented in *FindAllMarkers* from Seurat, with p values corrected by the false discovery rate (FDR) procedure (**Tables S10–12**).

​

### Analysis of reference transcriptomes from T CD4 populations in the Database of Immune Cell Expression (DICE)

Pre-processed RNA-Seq data from purified CD4 T populations of 91 healthy donors were downloaded from the DICEdb webportal (31) on May 2023. We analyzed a total of 519 samples including 88 Th1 (CD3^+^CD4^+^CD45RA^-^CD25^low^CD127^high^CXCR3^+^CCR4^-^CCR6^-^), 80 Th2 (CD3^+^CD4^+^CD45RA^-^CD25^low^CD127^high^CXCR3^-^CCR4^+^CCR6^-^), 88 Th17 (CD3^+^CD4^+^CD45RA^-^CD25^low^CD127^high^CXCR3^-^CCR4^+^CCR6^+^), 87 Th1/Th17 (CD3^+^CD4^+^CD45RA^-^CD25^low^CD127^high^CXCR3^+^CCR4^-^CCR6^+^) and 176 naive/memory Treg (CD3^+^CD4^+^CD45RA^+^CD25^high^CD127^low^ and CD3^+^CD4^+^CD45RA^-^CD25^high^CD127^low^) subsets. We compared samples from each subset to all other samples using two-sided t test, and corrected p values using the FDR procedure. For each subset, we defined a signature as those genes with a log2(fold change) > 1 and an adjusted p value < 0.05 (**Table S13**).

### Identification of cytotoxic CD4 T cells in additional scRNA-Seq datasets

We collected publicly available scRNA-Seq data from T cells found in colonic mucosa of patients with active UC (Mitsialis et al., 2020 (32), GSE150115) and inflamed ileum of CD patients (Martin et al., 2019 (33), GSE134809). We removed low quality cells with less than 500 genes detected and less than 10% of counts mapping to mitochondrial genes. Total UMI counts per cell were normalized to ten thousand (CP10K) and log(CP10K + 1) transformed. Clustering analysis was performed with the Seurat package, as described for our in-house scRNA-Seq data, to segregate T CD8, T CD4conv, Tregs and cycling T cells. Subtypes were annotated according to the expression of *CD8A, CD4, FOXP3, IL2RA* and *MKI67*. Cytotoxic CD4conv T cells were defined as those co-expressing *GZMA*, *GZMB*, *GNLY*, *IFNG*, *PRF1*.

### Calculation of gene signature scores within single cells

To score the expression of a given gene signature in individual cells using scRNA-Seq data, we first mean-centered (*Er*) scaled expression values per gene across all cells in a given dataset (*Eri,j* = *Ei,j* – avg(*Ei,1…n*), for gene *i* in cell *j*). Scores were then defined for each cell as the mean *Er* of genes in the signature. We analyzed manually defined signatures composed of classical, well-described markers of cytotoxicity (*GZMA*, *GZMB*, *GNLY*, *IFNG* and *PRF1)*, Th1 cells (*TNF*, *STAT1*, *STAT4*, *CXCR3* and *IFNG*) and Th17 cells (*CCR6*, *STAT3*, *RORA*, *IL17A*, *RORC*), as well as signatures generated by analyzing RNA-Seq data from Th1, Th2, Th17, Th1/Th17 and Treg samples retrieved from DICEdb (**Table S13**).

### Evaluation of cytotoxic CD4 T cells in IBD and control samples

We collected publicly available processed RNA-Seq data from whole tissue samples of 47 IBDs and 42 normal mucosa collected from areas adjacent to colorectal tumors (GSE166925 (34)). Samples were classified by pathological examination as either macroscopically active inflamed or noninflamed sites. Histologic (microscopic) inflammation was also evaluated according to the Nancy index using formalin-fixed paraffin-embedded tissue sections stained with hematoxylin and eosin. To estimate the abundance of cytotoxic CD4 T cells in the tissues analyzed, we first normalized gene expression data to control for potential signals derived from CD8 T cells. TPM expression values of each sample were multiplied by the relative proportion of CD4 T cells predicted by CIBERSORT with the LM22 reference mixture. In this step, three samples had a deconvolution p value > 0.05 and were excluded. Normalized expression values were log2(x + 1) transformed and mean-centered by gene across samples (*Eri,j* = *Ei,j* – avg(*Ei,1…n*), for gene *i* in samples *j*). The final score was then defined as the mean *Er* of genes with significantly increased expression in cytotoxic CD4 T cells detected in our *in-house* scRNA-Seq experiment (**Table S12**).

## Results

### Characterization of the chromatin accessibility of CD4 T cells in IBD patients

To investigate how chromatin accessibility may differ between IBD-affected tissue from inflamed and non-inflamed regions, we performed ATAC-Seq on sorted CD4 T cells isolated from IBD-affected individuals (**Tables S1, 2**). The degree of macroscopic tissue inflammation was classified by endoscopic score of the segment in which the sample was taken. Saturation analysis predicted that over 95% of all potentially identifiable ATAC-Seq peaks had been called in each biopsy condition, and at least 80% in the IBD blood and healthy control blood groups (**Figures S2A–D**). We identified an average of approximately 30,000 regions of accessible chromatin in inflamed biopsy samples and 20,000 regions in non-inflamed biopsy samples (**Figure S3A**). We also performed ATAC-Seq on CD4 T cells from the peripheral blood of 9 healthy controls and 25 IBD patients (**Table S2**). An average of approximately 27,000 peaks of chromatin accessibility were identified in healthy blood samples, relative to an average closer to 20,000 in IBD blood samples (**Figure S3A**). Overall, 84,782 unique peaks were identified in the gut-derived samples and 113,868 unique peaks in the blood-derived samples. We first performed dimensionality reduction over the maximum fold enrichment of signal over background, for each region of chromatin accessibility reported in at least one sample, using the UMAP algorithm. This revealed that the samples were primarily clustered by whether they were derived from a biopsy or blood, with inflammation or IBD status not driving the global clustering (**Figure S3B**).

**Figure 1:**
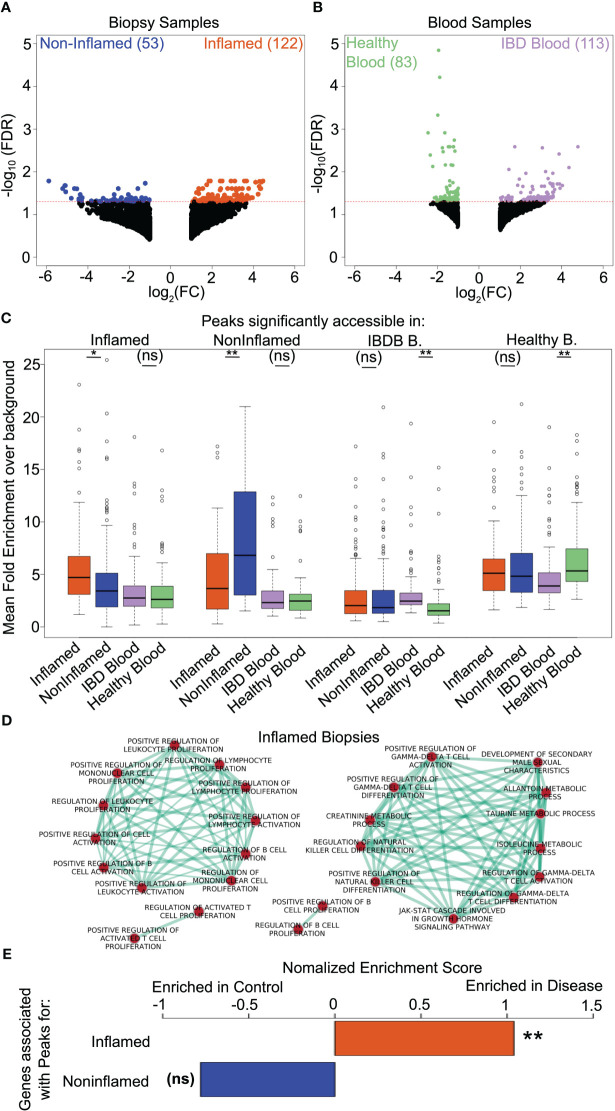
A subset of chromatin accessibility changes is localized to the site of inflammation in IBD patients. **(A, B)** Volcano plot showing log_2_ fold change (x axis) vs -log_10_(FDR) of chromatin accessibility (FDR corrected, negative binomial test over read counts computed using EdgeR for all sites with a minimum of log 2-fold change between conditions) for a) CD4 T cells from Inflamed (orange) vs non-inflamed (blue) biopsies and b) CD4 T cells from the peripheral blood of IBD (purple) vs healthy (green) patients. Dotted red line indicates a q value of 0.05, non-significant changes are shown as black dots. **(C)** Boxplots showing the mean fold enrichment over background for the significantly differentially accessible peaks in each condition within each other condition, colours as per volcano plots. Differential accessibility between all groups was assessed using the Wilcoxson sign rank test. **(D)** Genes regulated by differentially accessible regions in CD4 T cells from non-inflamed and inflamed biopsies were predicted as per Thurman et al. (29). Enrichment analyses of GO biological process on such predicted genes were performed using the BINGO plugin in Cytoscape. Shown are the inflammatory pathways enriched in genes regulated by regions that were more accessible in CD4 T cells from inflamed biopsies. **(E)** Normalized enrichment scores comparing the expression of genes that were more accessible in CD4 T cells from inflamed (top bar) or noninflamed (bottom bar) biopsies in CD4 T cells isolated from IBD patients vs. those isolated from age-matched controls. Scores were calculated using GSEA. *p<0.05; **p<0.01; ns: non-significant.

We found that 122 sites were significantly more accessible in CD4 T cells from inflamed biopsies, whereas 53 sites were more accessible in the CD4 T cells from non-inflamed biopsies (**Figure 1A**). Among the blood samples, 113 sites were more accessible in peripheral CD4 T cells from IBD patients compared with 83 sites that were more accessible in peripheral CD4 T cells from healthy donors (**Figure 1B**). Interestingly, none of the differentially accessible regions identified in CD4 T cells from tissue biopsies were detected as differentially accessible in peripheral blood CD4 T cells and vice-versa.

Next, we examined the distribution of the mean fold change of signal over background for each sample at the differentially accessible regions. We found that differentially accessible chromatin regions in biopsy samples were usually also accessible in blood, but no significant differences between the IBD and the healthy control blood samples were observed (**Figure 1C**). Similarly, regions significantly more accessible in IBD blood compared to healthy controls were also accessible in biopsy samples regardless of inflammation status, whereas regions significantly more accessible in healthy control blood compared to IBD patient blood showed similar levels of accessibility to the IBD patient biopsies (**Figure 1C**).

Overall, we found 116 genes potentially regulated by accessible regions identified in non-inflamed biopsies and 271 in inflamed biopsies (**Table S6**). *CD38*, *CXCL13*, *TNFRSF18* and *MYD88* are examples of several notable pro-inflammatory genes regulated by open regions in CD4 T cells from inflamed biopsies (**Table S6**). We also performed Gene Ontology (GO) enrichment analysis (35) on these genes associated with inflamed biopsies. In CD4 T cells from inflamed biopsies, most of the significantly enriched GO categories were related to activation, differentiation, proliferation, or regulation of specialized lymphocytes, such as γδT, NK, T and B cells (**Figure 1D**). On the other hand, GO terms for genes associated with CD4 T cells from non-inflamed biopsies or blood samples were not enriched for inflammatory pathways, suggesting that chromatin accessibility profiles within CD4 T cells from non-inflamed intestinal biopsies or from peripheral blood are unlikely to contribute to gut inflammation.

To determine whether the differences in chromatin accessibility identified in CD4 T cells from the biopsies were affecting the expression of genes important for the development of IBD, we compared our results to an RNA-Seq dataset from CD4 T cells isolated from gut biopsies of 21 CD patients and 12 age/sex-matched healthy controls (36). Using GSEA (35), we observed that genes associated with T cell ATAC-Seq peaks from inflamed biopsies had a significant enrichment within RNA-Seq data from the IBD patient samples, whereas genes associated with ATAC-Seq peaks from non-inflamed biopsies were more comparable to control samples (**Figure 1E**). Taken together, this suggests that a set of inflammation-associated, tissue-specific chromatin accessibility changes is associated with significant gene expression differences in IBD.

### Chromatin accessibility changes reveal a higher predominance of pTh17 cells in inflamed intestinal sites of IBD patients

To evaluate whether the inflamed-specific chromatin sites are more associated with specific CD4 T cell subpopulations, we compared these sites against the accessible chromatin regions specific to highly resolved CD4 T cell subtypes relative to naive CD4 T cells. Naive CD4 T cells were isolated from peripheral blood of healthy human volunteers (**Table S3**) and *in vitro* polarized into Treg-like, Th1, Th2, pTh17, and rTh17 cells. ATAC-Seq was performed on these subpopulations, as well as on effector and naive CD4 T cells. Any accessible region called in at least 3 replicates of each CD4 T cell subpopulation, but not in the effector CD4 T cells, was considered a site gained during polarization (**Table S7**). Similarly, ATAC-Seq sites lost in at least 3 replicates relative to effector CD4 T cells were considered lost during polarization (**Table S7**). After cell polarization, CD4 T cell subsets showed high enrichment for chromatin accessibility for known key lineage molecules (pTh17 cells: *RORC*, *RORB*, *IL6R*, *STAT3*, *IL22*, *BATF3*, *GZMB*, *GZMK*, *STAT4*, members of the IFN and TNF families and *THADA*), (rTh17: *IL23R*, *CCR6*, *RORB*, *IL21R* and *IL10*), (Th1: *STAT4*, *IFNG, IFNE*, *RELA*, *RELB*, *IL12RB*), (Th2: *GATA3*, *GATAD2B* and *IL10*) and (Tregs: *STAT5B*, *IL10*, *IL27*, *FOXR1*, *FOXP1*, *FOXK1* and *FOXK2*).

**Figure 2:**
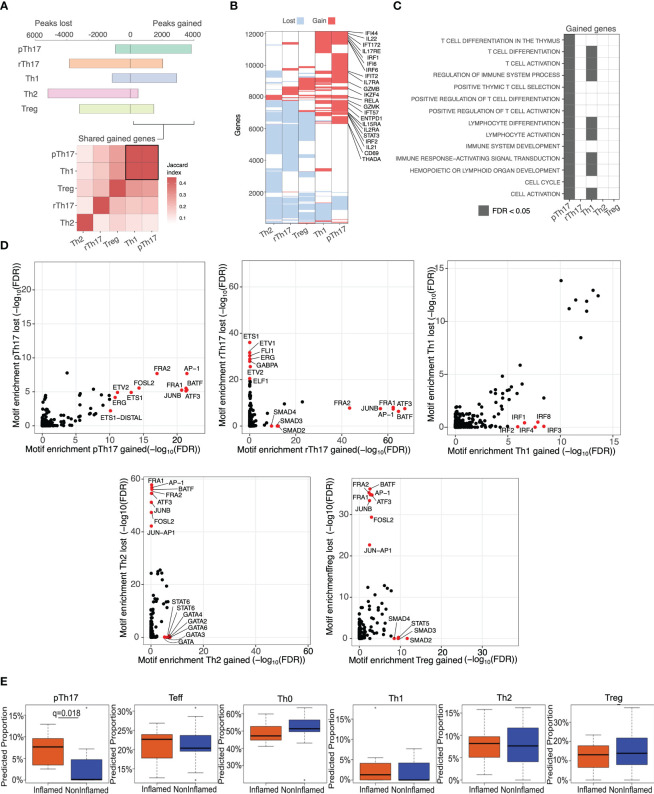
Chromatin accessibility changes associated with pTh17 T-Cell polarization are associated with inflamed biopsy-specific gains of chromatin accessibility. **(A)** Top: Number of accessible sites gained (right) or lost (left) following polarization into different CD4 T cell subpopulations relative to naïve CD4 T cells. Sites considered as gained/lost during polarization were detected in at least 2/3 of samples of each given condition and excluded those detected in effector CD4 T cell samples. Bottom: Pairwise similarity between set of genes gained by each cell type, ordered by hierarchical clustering. **(B)** Genes that were gained/lost by each cell type. Genes and cell types were ordered by hierarchical clustering. Relevant genes gained by both Th1 and pTh17 cells were annotated. **(C)** Enrichment analysis of GO biological process on gained genes in each T cell population. Significantly enriched terms (FDR < 0.05) are shown in gray. **(D)** Motifs enriched within subpopulation specific peaks identified by Homer with default parameters, using as background the catalog of peaks called in at least one sample. Relevant motifs that were significantly enriched (FDR < 0.05) are shown in red. **(E)** Boxplots of predicted proportions of each population within each inflamed (orange) and non-inflamed (blue) biopsy based on CIBERSORT deconvolution at the subpopulation-specific peaks. Significance computed via Wilcoxson sign-rank test.

Overall, pTh17 and Th1 cells showed a bias towards gain of chromatin accessibility, whereas rTh17, Th2 and Treg-like populations showed a bias towards loss of chromatin accessibility (**Figure 2A**, top). Interestingly, when peaks gained across all T cell subpopulations were compared, we found a higher resemblance between peaks gained by Th1 and pTh17 cells relative to the peaks gained by other T cell subpopulations (**Figure 2A**, bottom).

To better understand which processes may be modulated by such alterations of chromatin accessibility, we annotated the genes associated with the gain/loss sites in each T cell subpopulation (**Figure 2B**). Notably, pro-inflammatory pTh17 and Th1 cells showed the higher proportions of shared genes associated with gained ATAC-Seq peaks (**Figure 2B**). Further, GO enrichment analyses revealed that the genes associated with peaks gained in Th1 and Th17 cells, but not in other CD4 subpopulations, were enriched for immune activating programs, including lymphocyte and T cell activation and differentiation and immune response-activating signal transduction (**Figure 2C**).

Next, we examined the enrichment of DNA recognition motif sequences among sites that were gained/lost by each T cell subpopulation (**Table S9**). Relative to a background of all ATAC-peaks called in any population, DNA recognition motif enrichment analysis showed that the regions gaining chromatin accessibility intensity were enriched for known lineage-specific DNA recognition motif families (**Figure 2D**). As expected, motif analysis in regions gained by pTh17 cells revealed enrichment of pro-inflammatory-related TF motif sequences, such as BATF, AP-1, FRA1/2, JUNB and ATF3. The TF motif sequences enriched in pTh17 cells were also found in rTh17 cells, however the motif enrichment analysis also revealed an enrichment of anti-inflammatory TFs, such as SMAD2/3/4, within rTh17 cells. In addition, a gain in accessibility of IRFs (IRF1, 2, 3, 4 and 8) was exclusively found in Th1 cells. Members of the GATA family (GATA2, 3, 4 and 6) and STAT6 binding sites were enriched in regions gained by Th2 cells. Finally, motif analysis showed a gain in accessibility of STAT5 and SMAD2, 3 and 4 sequence motifs in Treg cells (**Table S9**, **Figure 2D**). These data further support the relevance of our CD4 T cell subtypes and support a relationship between changes in the chromatin accessibility and known biology associated with each subtype.

Next, we aimed to determine whether the regions preferentially accessible in T helper cell subpopulations were differentially accessible in the CD4 T cells from inflamed and non-inflamed tissues of IBD patients. To this end, we developed a method to predict the relative contribution of accessible chromatin specific to different CD4 T cells subsets in the IBD biopsies analyzed. First, based on the accessible elements unique to each CD4 T cell subtype (**Table S8**), we generated a set of synthetic test datasets based around randomly selected proportions of each population. We next tested whether the CIBERSORT algorithm (37) was able to predict the composition of the synthetic populations [as per Corces et al. (38)]. In all cases, the CIBERSORT deconvolution p value was <0.05 and the predicted error was <1%, which was set as our error threshold (**Figure S4**). Having validated this approach on synthetic data, we next applied it to our ATAC-Seq data from inflamed and non-inflamed biopsies. The predicted proportions of rTh17 cells were omitted as in most cases it was below our error threshold of 1%, suggesting that this population is scarce in intestinal tissue of IBD patients. We predict a significantly higher contribution of pTh17-specific accessible chromatin to the overall signal observed in CD4 T cells from inflamed biopsies compared to the non-inflamed biopsies (**Figure 2E**). We also found an increase in the frequency of effector and Th1 cells in inflamed biopsies relative to non-inflamed biopsies. On the other hand, the frequency of other T helper cell populations was similar between inflamed or non-inflamed biopsies (**Figure 2E**).

Taken together, these results suggest that the chromatin accessibility gains observed in the inflamed samples may be attributed to an increase in the pTh17 population. In addition, it is noteworthy the resemblance in terms of chromatin accessibility between pTh17 and Th1 cell subpopulations relative to the other subpopulations, shown by the higher overlap between peak-associated genes gained by pTh17 and Th1 cells (**Figures 2A, B**) as well as the enrichment of inflammatory pathways of genes associated with peaks gained by each CD4 T cell subpopulation (**Figure 2C**), further suggesting that pTh17 cells express Th1 markers.

### IBD risk loci are enriched within accessible chromatin regions specific to intestinal-derived CD4 T cells and pTh17 cell population

**Figure 3:**
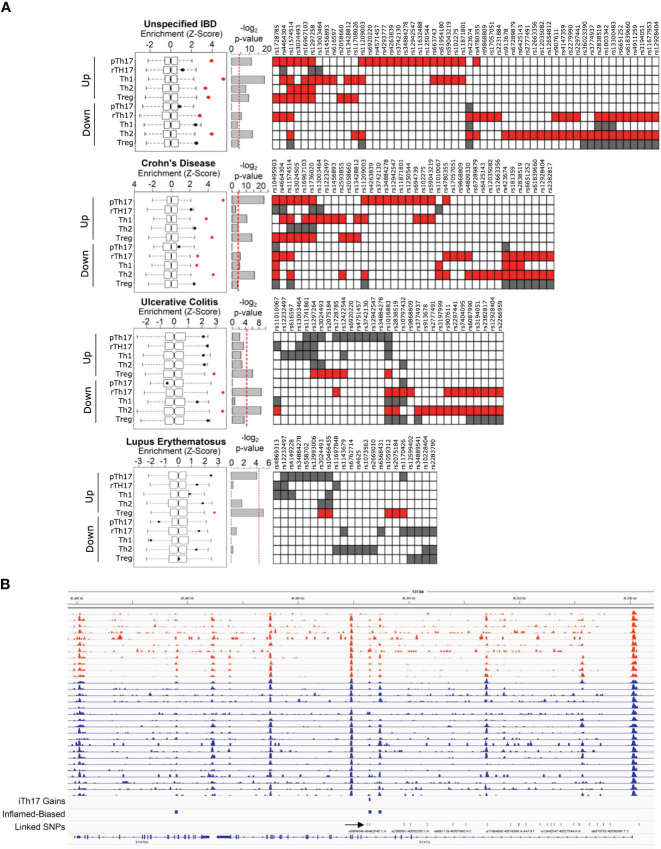
IBD risk loci are enriched within accessible chromatin regions in intestinal-resident CD4 T cells and pTh17 cell population. **(A)** Enrichment of all risk SNPs for IBD, Crohn’s Disease, Ulcerative Colitis and Lupus Erythematosus in the NGHRI GWAS catalogue. (right) Box and whisker plots show the enrichment score distribution of risk SNPs within the matching null set for each set of accessible chromatin loci unique to each subpopulation relative to effector CD4 T cells. The bar inside the box corresponds to the median enrichment score of the null set. Points correspond to observed enrichment. The significantly enriched genome regions (Bonferroni corrected *P*-value < 0.01) are marked in red. (center) bar plots show -log_2_ P-value for each enrichment. (right) Heatmaps show which annotated risk SNPs fall into an accessible region in any condition. White indicates the SNP is not present in this set of loci, red and black indicate presence of that SNP in that particular set of loci with red indicating that the set of risk SNPs are enriched in that condition. **(B)** ATAC-Seq signal profile of a Th17-related region, namely STAT3 intron, enriched in inflamed biopsies (orange) relative to non-inflamed biopsies (blue), where an IBD risk SNP (rs12942547) lies within (indicated by an arrow).

Interestingly, most of the differentially accessible regions of CD4 T cells from tissue samples and *in vitro* polarized CD4 T cell subpopulations are within intergenic or intronic regions of the genome (**Figure S5**). As it has been shown that approximately 80% of IBD risk loci also fall within intergenic or intronic regions of the genome (3), we analyzed whether IBD risk loci were enriched within the differentially accessible regions of CD4 T cells from tissue biopsies and *in vitro* polarized CD4 T cell subpopulations. By using Variant Set Enrichment (VSE) analysis, we found that several IBD risk loci were enriched within the differentially accessible regions of CD4 T cells from inflamed biopsies and the regions of chromatin accessibility gained in Th1 and pTh17 subpopulations (**Figure 3A**). For comparison, we analyzed a set of risk SNPs associated with systemic lupus erythematosus (SLE), an autoimmune disease whose outcome is less dependent on pro-inflammatory CD4 T cells than IBD. This set of SLE risk loci were also enriched within differentially accessible regions from *in vitro* polarized Treg-like cells. However, we did not find any significant enrichment of SLE risk loci within the accessible regions of pro-inflammatory CD4 T cell subpopulations (**Figure 3A**). The functional annotation of IBD risk SNPs and their proximity with gene targets within the genome can be found at **Table S14**. Strikingly, many IBD risk loci were enriched within genomic pTh17-associated regions from inflamed biopsies, such as an intronic area near *STAT3* (**Figure 3B**). These genetic variants were mostly CD risk loci rather than UC risk loci. These findings align with the reported association between Th1/Th17 programs with CD (14, 39). Regions near known pTh17-related genes, such as *IL23R*, and regions near other genes, such as *ATG16L1*, *ANKRD55* and *THADA*, are also highly enriched in both pTh17 cells and inflamed biopsies relative to other CD4 T cell subpopulations and non-inflamed biopsies, respectively, and where IBD-risk SNPs fall within (**Figure S6**).

### Single cell transcriptomics reveals that CD4 T cells co-expressing Th1, Th17 and cytotoxic gene programs are enriched in IBD inflamed tissues

**Figure 4:**
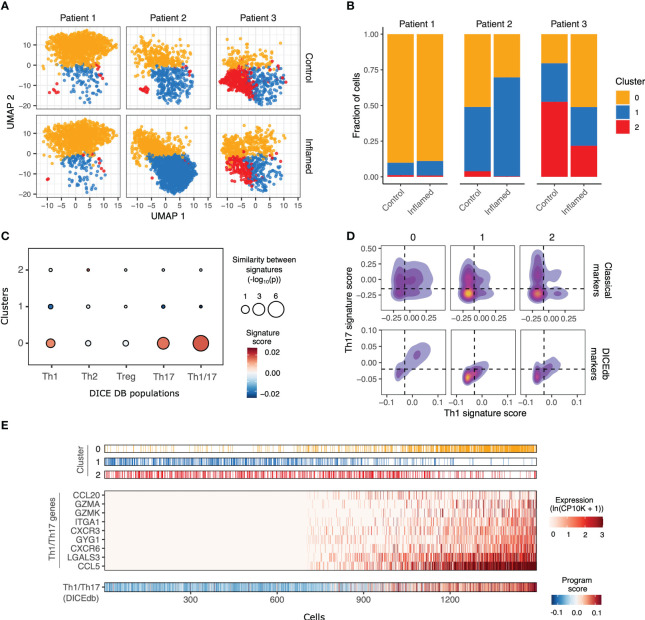
Single cell transcriptomics reveals a high proportion of cytotoxic pTh17-like cells in inflamed intestinal sites of Crohn’s disease patients. **(A)** UMAP plots of CD4 T cells from control and inflamed samples across 3 IBD patients, colored based on Louvain clustering (C0 – C2). **(B)** Bar chart showing the proportion of cells from each sample mapping to each cluster. **(C)** Bubble plot depicts the similarity between C0-C2 cells and reference T CD4 populations from the DICEdb. Bubble size is proportional to the significance of overlap (hypergeometric test) between gene signatures from C0-C2 and DICEdb populations. Bubbles are colored according to the average expression of DICEdb signatures by cell in C0-C2. **(D)** Density plots showing the expression of Th1 and Th17 gene signatures by cells in C0-C2. Shown are both classical signatures (Th1: *TNF*, *STAT1*, *STAT4*, *CXCR3* and *IFNG;* Th17: *CCR6*, *STAT3*, *RORA*, *IL17A*, *RORC*) as well as those derived from the DICEdb populations. **(E)** Expression of DICEdb Th1/Th17 genes by C0-C2 cells. We only included genes with significantly increased expression in C0. Cells are ordered by their Th1/Th17 scores, as shown in the lower panel. Bar on the bottom shows the expression of the complete Th1/Th17 signature by each cell. Bars on top annotate the cluster of each cell.

To better understand the heterogeneity of CD4 T cells from tissue samples and the transcriptional program of pTh17 cells in IBD patients, we generated single cell RNA-Seq profiles for 5,902 CD4 T cells from matched non-inflamed and inflamed biopsies from three CD patients (**Tables S4, S5**). Louvain clustering of cells identified three main subsets, which were found in all three patients (**Figure 4A**) but in different proportions (**Figure 4B**). C0 cells showed a hybrid phenotype (**Figure 4C**, **Table S10**), with increased expression of both Th1 (e.g., *CXCR6*) and Th17 (e.g., *CCL20*) markers, while C2 cells expressed *CCR7*, *FOS*, *JUN*, *CD69* and *CXCR4* (**Figure 4C**, **Table S10**), suggestive of memory CD4 T cells.

To confidently annotate these cell clusters, we compared their expression profiles to those of well-defined CD4 T cell subpopulations (Th1, Th2, Th17, Th1/17, and Treg) from the DICEdb (31) (**Table S13**). Importantly, we found that C0 cells share features with Th1 and Th17 cells and are especially similar to the Th1/17 subset (**Figure 4C**). Notably, this cluster had the highest frequency of cells co-expressing Th1 and Th17 gene signatures, both when considering the classical markers for these populations (Th1: *TNF*, *STAT1*, *STAT4*, *CXCR3*, *IFNG*; Th17: *CCR6*, *STAT3*, *RORA*, *RORC*, *IL17A*) as well as signatures derived from DICEdb (**Figure 4D**), and highly expressed genes derived from Th1/Th17 signature from DICEdb, such as *CCL20*, *GZMA*, *GZMK*, *ITGA1*, *CXCR3*, *GYG1*, *CXCR6*, *LGALS3* and *CCL5* (**Figure 4E**).

**Figure 5:**
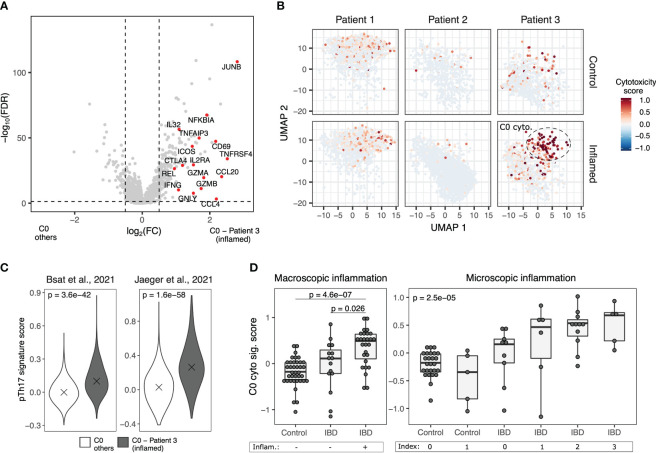
A subset of pTh17 cells has features of cytotoxicity. **(A)** Volcano plot showing differentially expressed genes between cells in the C0 cluster of the inflamed biopsy of patient 3, the most inflamed sample analyzed, and all other C0 cells. Dashed lines denote an FDR value of 0.05 and an absolute log2(fold change) of 0.5. **(B)** Expression of UMAP plots with cells colored by cytotoxic score. C0 cytotoxic cells observed in the inflamed biopsy of patient 3 are indicated. **(C)** Expression of two pTh17 gene signatures (40, 41) by cells in the C0 cluster of the inflamed biopsy of patient 3 compared to all other C0 cells. **(D)** Estimated abundance of C0 cytotoxic cells highlighted in **(B)** in normal mucosa and IBD samples with different degrees of inflammation [Friedrich M et al., 2021 (34)]. Comparisons in **(A, C** and **D)** (left) were performed using two-sided t test. Comparisons in D were performed using one-way ANOVA. P values in **(A)** were corrected using the FDR procedure.

Of the three patients sequenced using scRNA-Seq, patient #3 had a remarkably high endoscopic inflammation score. Thus, we compared the expression profile of C0 cells in the inflamed biopsy of patient #3 to C0 cells from all the other samples and found that they upregulate the expression of cytotoxic genes such as *GZMA*, *GZMB*, *GNLY*, *NKG7* and *IFNG* (**Figure 5A** , **Table S11**). Pathogenic Th17 cells showing co-expression of Th1 and cytotoxic signatures were also identified by other studies both in IELs (40) and mediastinal lymph nodes (41) of IBD patients. When applying the gene signature from these cells to C0 cells from our study, an enrichment was evident in cells from patient #3 (**Figures 5B, C**). Altogether, these results reveal that cells co-expressing Th1, Th17 and cytotoxic gene signatures are associated with IBD.

**Figure 6:**
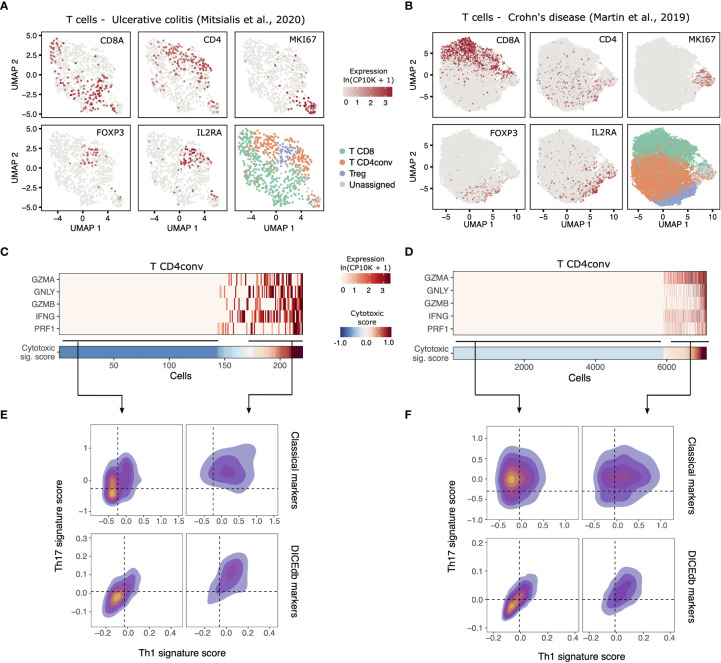
Identification of cytotoxic CD4 T cells in additional scRNA-seq datasets. **(A, B)** Identification of CD4conv T cells in colonic mucosa sample of patients with active UC (n= 195 cells, Mitsialis et al., 2020 [32)] and inflamed ileum samples of CD patients [n= 805 cells, Martin et al., 2019 (33)]. **(C, D)** Expression of cytotoxicity-related genes by CD4conv T cells in each dataset. Cells are ordered according to their cytotoxic scores, as shown in the lower panel. **(E, F)** Density plots showing the expression of Th1 and Th17 gene signatures by cells in C0-C2. Shown are both classical signatures (Th1: *TNF*, *STAT1*, *STAT4*, *CXCR3* and *IFNG;* Th17: *CCR6*, *STAT3*, *RORA*, *IL17A*, *RORC*) as well as those derived from the DICEdb populations.

To further confirm the clinical relevance of our findings in an independent cohort, we next analyzed published RNA-Seq data from whole tissue samples of 42 control mucosa specimens and 47 IBD with different degrees of disease severity (34). Notably, the estimated abundance of cytotoxic CD4 T cells was higher in macroscopically inflamed IBD samples compared to non-inflamed ones and controls (**Figure 5D**, left), and increased with the degree of microscopic inflammation (**Figure 5D**, right). In line with that, we identified subsets of cytotoxic CD4 T cells with a hybrid Th1/Th17 phenotype in scRNA-Seq data from two additional IBD studies (32, 33). Using unsupervised clustering, we segregated CD8 T cells, conventional CD4 T (T CD4conv) cells and Tregs from UC (**Figure 6A**) and CD (**Figure 6B**) patients. We then identified what T CD4conv cells were expressing or not a cytotoxic program (**Figures 6C, D**). Interestingly, cytotoxic CD4 T cells in both UC and CD samples had a higher rate of co-expression of Th1 and Th17 genes than those cells with no evidence of cytotoxicity (**Figures 6E, F**).

## Discussion

IBD is a complex disorder characterized by a dysregulated immune inflammatory response that affects the gastrointestinal tract (1). CD4 T cells are the main drivers of inflammation induction in both UC and CD (14, 26, 42). Although reports are emphatic about the importance of CD4 T cells to the disease (12, 13), the extent to which circulating and/or tissue-resident CD4 T cells represent unique populations that distinctly manage gut inflammation is still poorly understood. Our data suggest that CD4 T cells isolated from tissue samples, rather than circulating CD4 T cells, express a distinct chromatin accessibility profile associated with a network of inflammatory pathways in inflamed intestinal sites of IBD patients.

CD4 T cell subpopulations are different in UC and CD patients (14). However, the exact subpopulations involved in both diseases are still not fully understood, likely due to the averaging of cell populations using classical methods of analysis. Whereas CD seems to be predominantly induced by Th17 and Th1 cells, the fate of UC seems to be dictated by Th2 cells (14, 26, 42). Using the chromatin profiles of CD4 T cells cultured under Th1, Th2, Treg-like, rTh17 and pTh17 conditions, the chromatin accessibility changes revealed a higher predominance of pTh17 cells in inflamed intestinal sites of IBD patients, even though the cells used for profiling the chromatin accessibility of the T cell subsets were not presorted and were composed of an admixture of polarized and non-polarized CD4 T cells, which might have masked the identification of some known lineage-specific DNA recognition motif families. With the advent of single cell methodologies, the compositions of a given heterogeneous cell population can be better dissected into functional subsets. By analyzing the heterogeneity of CD4 T cells from IBD patients through single-cell transcriptomics, we identified a CD4 T cell subset, resembling pTh17 cells, that expresses a transcriptional program composed of Th1 and Th17 markers, which is enriched in inflamed areas of the intestine of UC and CD patients.

Specialized lymphocytes have the capacity of inducing inflammation by producing and secreting cytolytic granules. NK and CD8 T cells are the classical cell populations capable of inducing cell cytotoxicity. Upon recognition, NK (43) and CD8 T cells (44–46) form a cytotoxic immune synapse where cytolytic granules are released to kill target cells. In UC patients, it has been reported that *IL17A* can be expressed by CD4^+^, CD8^+^, and CD4^+^CD8^+^ T cells, while CD8^+^IL-17A^+^T cells can further express both cytotoxic and Th17 programs (47). Our results show that pTh17 cells from inflamed intestinal areas of IBD patients can also express a cytotoxic signature, thus suggesting that these cells could be linked with intestinal epithelial damage in IBD patients through a cytotoxic mechanism.

While holding great promise, our study has some limitations that need to be highlighted as following: i) our findings must be validated by functional *in vitro* assays and *in vivo* models, as a proof of concept that cytotoxic pTh17 cells can indeed target and damage intestinal epithelial cells. ii) This study lacks validation at the protein level of cytotoxic pTh17 cells in inflamed tissues of IBD patients. iii) Our cohort was not sufficiently large to segregate UC and CD patients when interrogating the chromatin accessibility of CD4 T cells; we then used all IBD samples (UC + CD) as a unique group. iv) The exclusion of CD4 T cells expressing CD161 (48) and/or Vα24Jα18 during the single cell RNA library preparation might have impacted the frequency of pTh17 cells in the inflamed intestinal sites of CD patients, which could explain why only patient #3 has presented cytotoxic pTh17 cells in their inflamed tissue.

IBD is thought to occur as a result of the complex interplay between host genetics and immune response. GWA studies have shown how critical the human genetic background is for the establishment of IBD and have identified genetic variants associated with IBD (3–7, 23, 25). Multiple variants have direct impact on the immune response and may aggravate the disease (3, 24, 26). CD4 T lymphocytes are a dynamic and plastic cell population, mediated in part, at the chromatin level (49–53). Our data highlight how disease-related regions of chromatin accessibility relate to transcriptional programs found in pTh17 cells from inflamed sites. Further validation in HaploReg and Regulome db showed that most of these variants hold putative functional impact on gene expression regulation. *In vitro* systems where single-nucleotide variants can be artificially inserted are needed to confirm the modulatory capacity of IBD risk SNPs on pTh17 cell differentiation.

Anti-IL-17A therapy has not proven successful in clinical trials with IBD patients (54, 55). Although our study has suggested a key role for pTh17 cells associated with intestinal inflammation of IBD patients, we believe that such inflammation goes beyond the solely production of IL-17A, and the accumulation of pTh17 cells within the inflammatory site might be associated with a very complex inflammatory network involving cytotoxic machinery. Thus, therapies blocking such cytotoxic machinery could be more effective to control the Th17 activity than the anti-IL-17A therapy. Furthermore, it is thought that a negative impact on the intestinal barrier function is a likely explanation for the lack of efficacy of IL-17 inhibitors in IBD (56).

Finally, chromatin and gene expression profiling of CD4 T cells linked inflammatory programs mainly composed of pTh17 cell features and cytotoxic molecules with inflamed areas of the intestine of IBD patients. Interestingly, IBD-risk SNPs were predicted to be enriched in accessible regions near the Th17-related genes *STAT3* and *IL23R* in both CD4 T cells from inflamed biopsies and pTh17 T cells. In summary, our data associate cytotoxic pTh17 with gut inflammation and link IBD risk variants with the chromatin accessibility of pTh17-related regions in IBD patients. Further studies are needed to investigate whether the cytotoxic pTh17 subpopulation can directly target and disrupt intestinal epithelial cells in IBD patients.

## Data availability statement

The datasets presented in this study can be found in online repositories. The names of the repository/repositories and accession number(s) can be found below: GSE226875 (GEO, RNA-seq), EGAD00001011066 (EGA, dataset), EGAS00001007343 (EGA, blood samples), EGAS00001007344 (EGA, biopsy samples), and EGAS00001007345 (EGA, in vitro-polarized CD4 T cell).

## Ethics statement

The studies involving human participants were reviewed and approved by Research Ethics Boards from the University Health Network (REB# 15-9499.2) and Mount Sinai Hospital (REB # 02-0234-E). The patients/participants provided their written informed consent to participate in this study.

## Author contributions

DC, CA, MaS and ML designed, supervised the research, and corrected the manuscript. TM performed all experiments, analyzed, and interpreted the data and wrote the manuscript. AM and GK analyzed and interpreted the ATAC-Seq data and wrote the manuscript. AK and GK analyzed and interpreted the single cell RNA sequencing results and wrote the manuscript. TM, AM, GK, GV, and AK prepared figures and tables. MiS and MaS derived all clinical metadata and supervised samples acquisition. MiS, WT, OS, WF, TM, AM, AK, DC, CA, MaS and ML contributed with discussions. HY contributed with samples acquisition.
