# Supplementary material for: The chromatin and single-cell transcriptional landscapes of CD4 T cells in inflammatory bowel disease link risk loci with a proinflammatory Th17 cell population
Source: Front Immunol. 2023 Aug 3;14:1161901. doi: 10.3389/fimmu.2023.1161901 (PMC10436103; doi:10.3389/fimmu.2023.1161901)
Supplement: Supplementary file 1 [file DataSheet_1.pdf]

Supplementary Material - Figures

The chromatin and single-cell transcriptional landscapes of CD4 T cells in inflammatory bowel disease link risk loci with a proinflammatory Th17 cell population

Tiago S. Medina[1,2#*], Alex Murison[1#], Michelle Smith[3], Gabriela S. Kinker[2], Ankur Chakravarthy[1], Glauco A. F. Vitiello[2], Williams Turpin[3], Shu Yi Shen[1], Helen L. Yau[1,4], Olga F. Sarmento[5], William Faubion[5], Mathieu Lupien[1,4], Mark S. Silverberg[3&*], Cheryl H. Arrowsmith[1,4,6&*], Daniel D. De Carvalho[1,4&*]

[1] Princess Margaret Cancer Centre, University Health Network, Toronto, Ontario, M5G 1L7, Canada
[2] International Research Center, A.C. Camargo Cancer Center, São Paulo, Brazil.
[3] Division of Gastroenterology, Mount Sinai Hospital, University of Toronto, Toronto, ON, Canada.
[4] Department of Medical Biophysics, University of Toronto, Toronto, Ontario, M5G 1L7, Canada
[5] Division of Gastroenterology and Hepatology, Mayo Clinic, Rochester, Minnesota, USA.
[6] Structural Genomics Consortium, University of Toronto, MaRS South Tower, Suite 700, 101 College Street, Toronto, Ontario M5G 1L7, Canada.

# These authors contributed equally.
& These authors contributed equally.

*** Corresponding authors: TSM, MSS, CHA, and DDC**
Address correspondences to
Dr. Daniel D. de Carvalho

SUPPLEMENTARY FIGURE 1

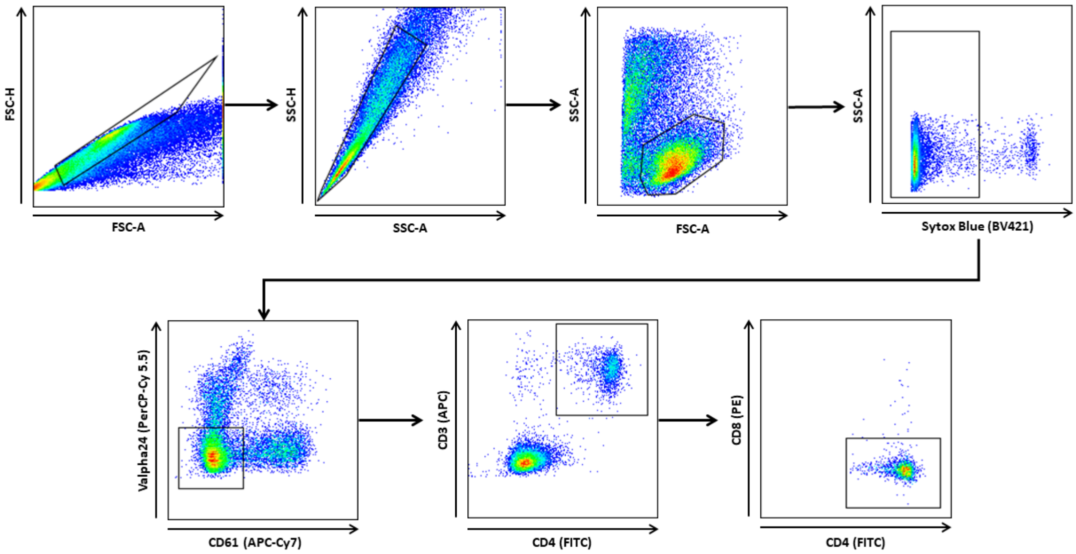

Fig S1. Gating strategy for the sorting of CD161^{-}Va24Ja18^{-}CD8^{-}CD4^{+} T cells from matched inflamed and non-inflamed biopsies of 3 CD patients. Sorted cells were used to prepare single cell RNA-Seq libraries of 5,902 CD4 T cells.

SUPPLEMENTARY FIGURE 2

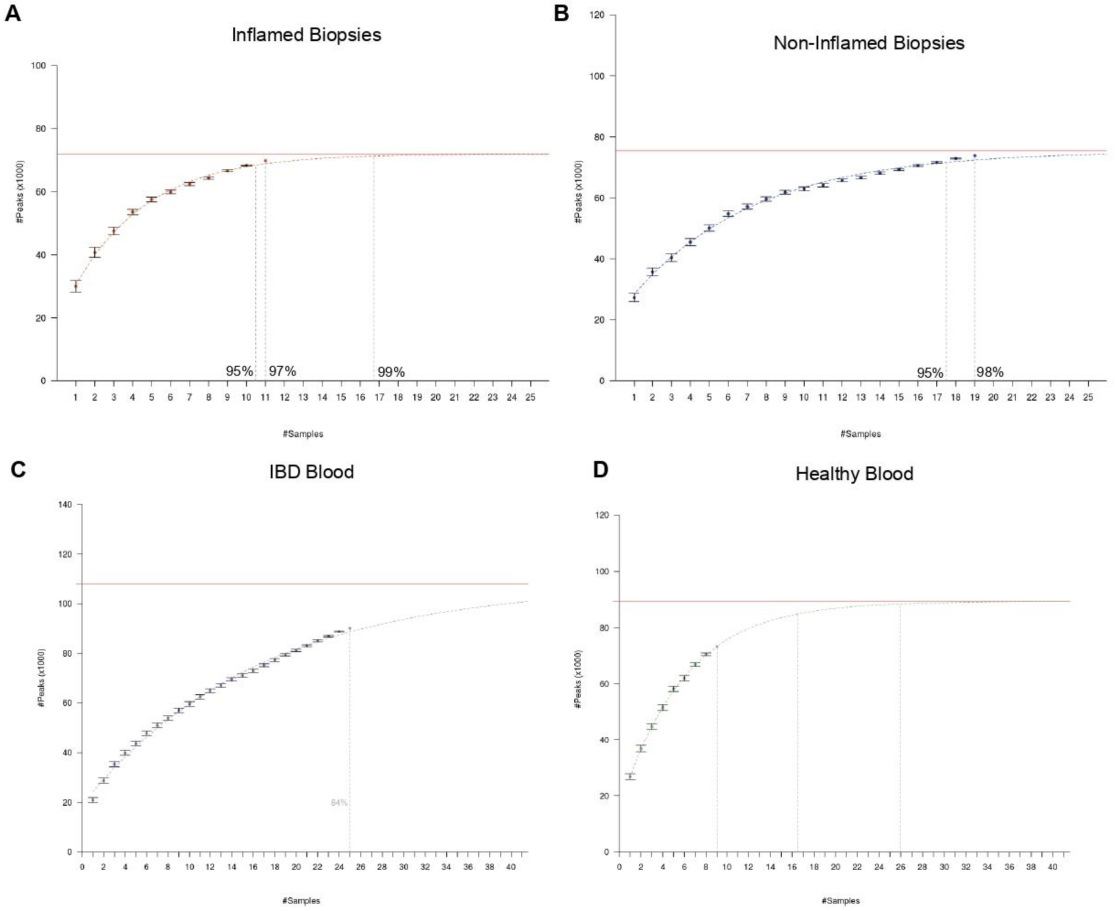

Fig S2. A non-linear regression model was fit to the number of new peaks obtained sequentially over all samples for 1,000 permutations of sample orders and stratified into whether they derived from **(A)** inflamed or **(B)** non-inflamed biopsies, as well as **(C)** IBD or **(D)** healthy blood. Individual points show the mean number of new peaks per sample added and associated standard error of the mean fitted blue line shows fitted model with saturation line (red) marked as well as estimated number of samples needed to identify 95, 97 and 99% of peaks (grey lines).

SUPPLEMENTARY FIGURE 3

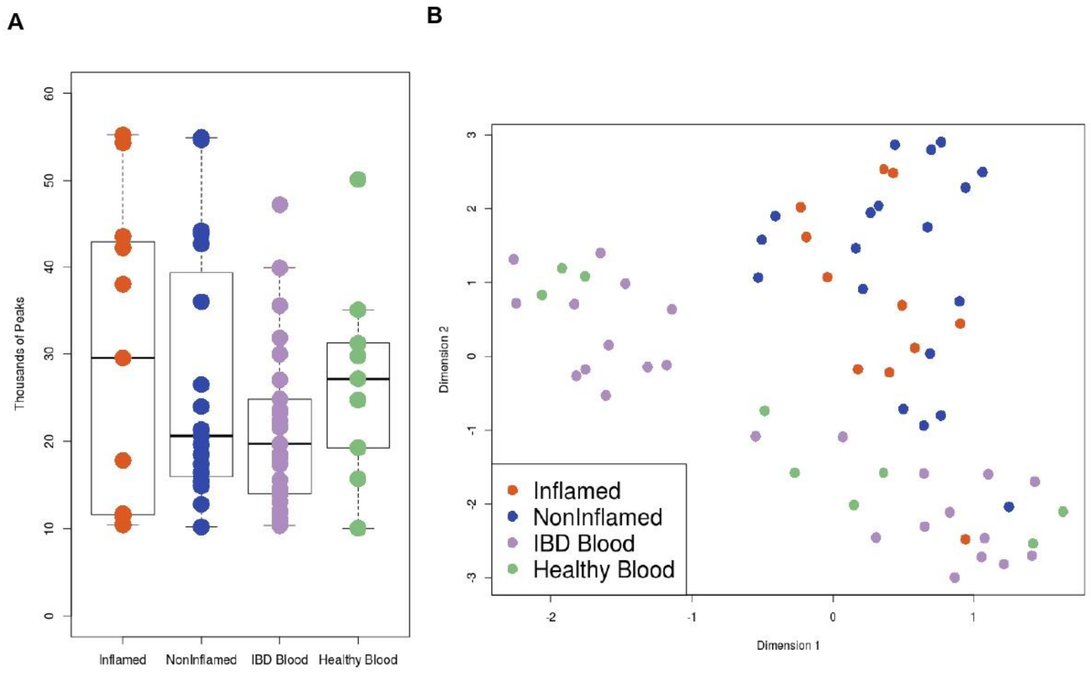

Fig S3. (A) Boxplots showing the number and distribution of peaks (in thousands) for the inflamed (orange) and non-inflamed (blue) biopsy ATAC-Seq samples, as well as the IBD Blood (purple) and healthy blood (green) ATAC-Seq samples. Whiskers indicate the upper and lower quartiles. **(B)** The first 2 dimensions of a principal component analysis between all ATAC-Seq samples for the quantile normalized maximum signal over background for each peak in the catalogue of called peaks.

SUPPLEMENTARY FIGURE 4

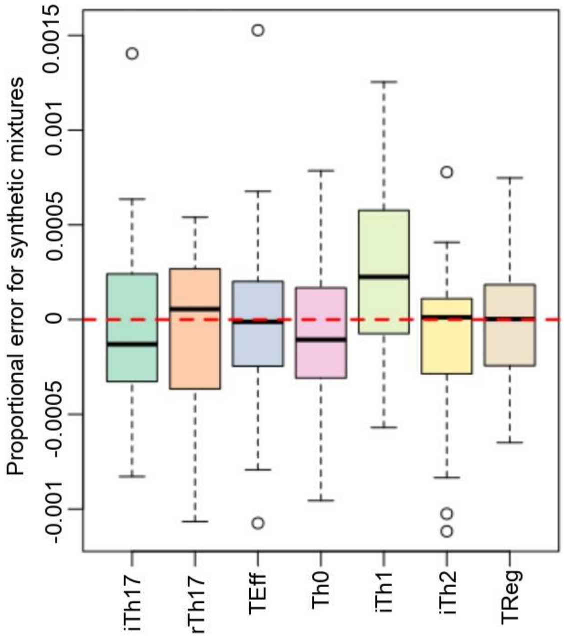

Fig S4. Boxplots indicate the distribution of difference between predicted proportion generated via CIBERSORT and the proportion to which that population contributed to the synthetic mixture. Whiskers indicate the upper and lower quartiles. Synthetic mixtures were generated as per Corces et al, 2016 (ref. 38 in the main text).

SUPPLEMENTARY FIGURE 5

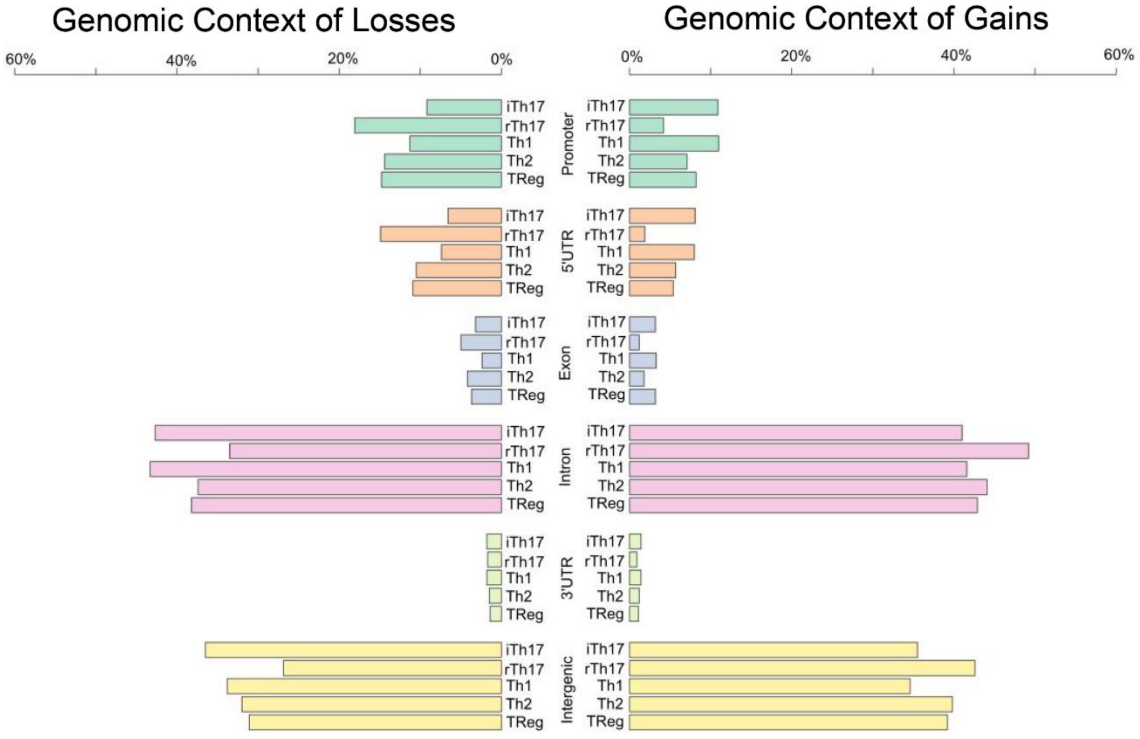

Fig S5. Genomic context of regions gained or lost across all CD4 T cell subpopulations relative to effector CD4 T cells distributed according to their genomic location into promoters, 5’ UTR, exons, introns, 3’ UTR or intergenic. Sites were called as present in each T cell subpopulation detected in at least 2/3 of samples.

SUPPLEMENTARY FIGURE 6

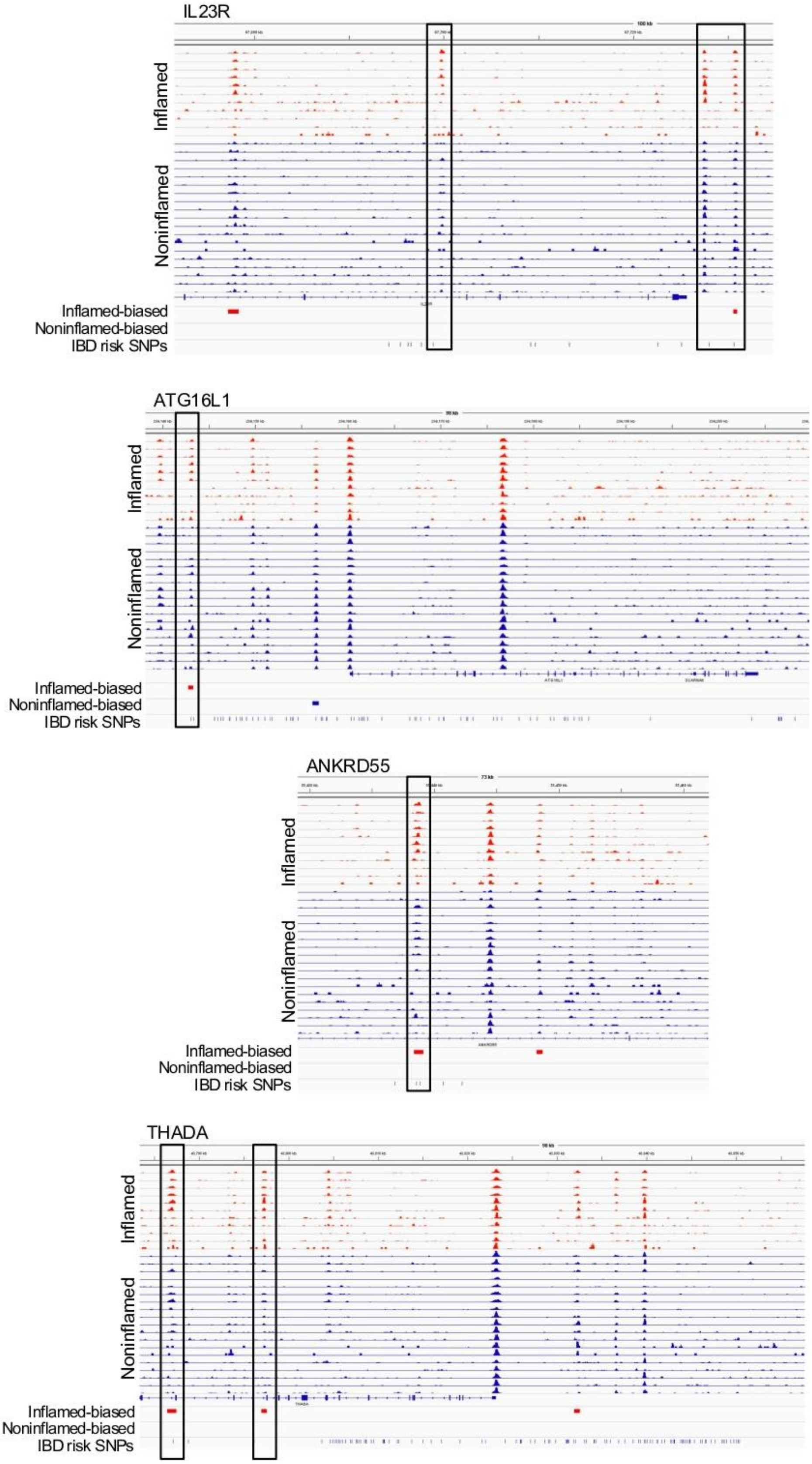

Fig S6. Other examples of ATAC-Seq signal profile from regions that are more accessible in CD4 T cells from inflamed biopsies relative to CD4 T cells from non-inflamed biopsies and where IBD-risk SNPs fall within (indicated by rectangles).
